# Influence of Partially Carboxylated Powdered Lignocellulose from Oat Straw on Technological and Strength Properties of Water-Swelling Rubber

**DOI:** 10.3390/polym16020282

**Published:** 2024-01-19

**Authors:** Elena Cherezova, Yulia Karaseva, Abdirakym Nakyp, Airat Nuriev, Bakytbek Islambekuly, Nurgali Akylbekov

**Affiliations:** 1 Department of Synthetic Rubber Technology, Institute of Polymers, Kazan National Research Technological University, 68 K. Marx Str., 420015 Kazan, Russia; cherezova59@mail.ru (E.C.); karaseva_j@mail.ru (Y.K.); aurat0@inbox.ru (A.N.); 2 Laboratory of Engineering Profile “Physical and Chemical Methods of Analysis”, Korkyt Ata Kyzylorda University, 29A, Aiteke bi Str., Kyzylorda 120000, Kazakhstan; nurgali_089@mail.ru

**Keywords:** flotoreagent oxal, powdered lignocellulose, carboxylation, nitrile butadiene rubber, swelling rubber, IR spectrum, microwave radiation

## Abstract

The work is aimed at the development of an energy-saving technique involving the partial carboxylation of powdered lignocellulose products from the straw of annual agricultural plants and the use of the obtained products in rubber compositions as a water-swelling filler. Lignocellulose powder from oat straw (composition: α-cellulose—77.0%, lignin—3.8%, resins and fats—1.8%) was used for carboxylation without preliminary separation into components. Microwave radiation was used to activate the carboxylation process. This reduced the reaction time by 2–3 times. The synthesized products were analyzed by IR spectroscopy, thermogravimetry and scanning electron microscopy. Industrial product sodium carboxymethylcellulose (Na-CMC) was used as a swelling filler for comparison. The swelling fillers were fractionated by the sieve method; particles with the size of 0–1 mm were used for filling rubber compounds. The amount of swelling filler was 150 parts per 100 parts of rubber (phr). Due to the high filling of rubber compounds, plasticizer Oxal T-92 was added to the composition of a number of samples to facilitate the processing and uniform distribution of ingredients. The rubber composition was prepared in two stages. In the first stage, ingredients without swelling filler were mixed with rubber on a laboratory two-roll mill to create a base rubber compound (BRC). In the second stage, the BRC was mixed with the swelling filler in a closed laboratory plasti-corder rubber mixer, the Brabender Plasti-Corder^®^ Lab-Station. Vulcanization was carried out at 160 °C. For the obtained samples, the physical-mechanical and sorption properties were determined. It has been shown that the carboxylated powdered lignocellulose from oat straw increases the strength properties of rubber in comparison with Na-CMC. It has been shown that when the carboxylated powdered lignocellulose from oat straw is introduced into the rubber composition, the degree of rubber swelling in aqueous solutions of various mineralizations increases by 50 and 100% in comparison with a noncarboxylated lignocellulose.

## 1. Introduction

Because of globalization and mass production, the amount of waste generated during the processing of cereals increases every year. Implementation of the Closed Cycle Economy Action Plan involves the integration of waste into the production cycle in order to obtain new useful products [1]. In this regard, the use of waste from annual cereal crops as a raw material base for obtaining valuable products for various industries has been an actual direction of scientific research for a number of years. The main component of leaves, bromes and stems of annual agricultural plants is cellulose with a small amount of lignin. The advantage of this type of raw material is annual renewability, ease of processing and low cost [2,3,4,5,6,7]. It helps to reduce the high dependence on petrochemical resources [8]. Lignocellulose has found new applications such as bioprocessing for the production of biofuels and biochemical products [9], pharmaceuticals [10] and multifunctional carbon materials [11,12].

In [13], lignocellulose powders obtained from waste cardboard were also used in rubber compositions as a modifier that increases the bond strength between vulcanized rubber and brass-plated steel cord, which allows for extension of the service life of products.

The ability of cellulose to absorb moisture well [14,15,16,17,18] allows for the use of lignocellulose raw materials as a filler in the creation of water-swelling polymer materials (PM), which have found application as sealing elements in building structures and sleeves for casing packers used in the oil and gas industry [19,20,21,22,23,24].

Na-CMC and its compositions with water-soluble polymers are most often used as water-swelling fillers in packer rubbers [25,26,27,28,29,30,31,32]. To ensure a high level of swelling, it is suggested to introduce Na-CMC in the amount of up to 200 parts per 100 parts of rubber. However, according to the literature data, the introduction of such an amount of water-swelling filler leads to an increase in the viscosity of the raw rubber compound and a decrease in the strength properties of the vulcanizate. In addition, over time, the water-soluble swelling filler is washed out of the polymer material, due to which the insulating function of the packer is lost [22,33]. The above shows the need to search for new fillers, which would provide long-term fluid isolation, a sufficient degree of swelling and the good strength characteristics of rubbers.

Previously, we have shown that the use of powdered lignocelluloses from grass crops can improve the strength characteristics of rubber; however, the ability of rubber to swell in aqueous media of different mineralization decreased [34,35,36].

The aim of the study was to increase the service life and degree of rubber swelling in aqueous media through partial carboxylation of lignocellulose from the straw of grass crops, in particular oat, while providing sufficient tensile strength characteristics of rubbers. Such raw materials have not previously been used in this process.

The scientific literature describes methods of carboxylation of cellulose by suspension and solid-phase methods. The described synthesis methods are sufficiently long in time [37,38]. In addition, in the described methods of cellulose carboxylation, a large amount of wastewater is generated. For activation of some chemical reactions occurring in polar medium, in a number of works, the use of microwave radiation is proposed [39]. This method allows for a reduction in reaction time and an increase in the intensity of the process, which significantly reduces energy costs [40].

An important aspect of the proposed process is the possibility of using production waste without preliminary separation into separate components. This allows us to significantly reduce costs due to the exclusion of additional stages of raw material preparation from the technological process.

## 2. Materials and Methods

### 2.1. Materials

The carboxylation reaction used powdered lignocellulose from oat straw (PC-Oat) (composition: α-cellulose—77.0%, lignin—3.8%, resins and fats—1.8%), obtained by [41]; NaOH (JSC “BASHKIR SODA COMPANY”, Sterlitamak, Russia), (pure for analysis, impurities not more than 1.0% by weight), isopropyl alcohol (JSC “ECOS-1”, Moscow, Russia) (chemically pure, impurities not more than 0.001% by weight), and monochloric acid (Nouryon, Delfzijl, The Netherlands) (chemically pure, impurities not more than 0.001% by weight), were used.

As a polymer base for creating sleeves for swelling packers, oil- and gasoline-resistant thermostable nitrile butadiene rubber (NBR) is widely used [42,43,44]; synthetic isoprene rubber, methyl-styrene-butadiene rubber, ethylene propylene rubber and their mixtures are used to a lesser extent [45,46,47]. In this work, nitrile butadiene rubber grade BNKS-28 AMN was used.

Swelling fillers with particle size 0.5–1.0 mm (SF) were used: Na-CMC (CJSC “Politsell”, Vladimir, Russia) from the Polycell CMC 9 brand, (degree of polymerization not less than 700, degree of substitution 0.8–0.9); oat straw powder (powdered cellulose from oat straw (PC-Oat)); partially carboxylated oat straw powder (carboxymethylcellulose from oat straw (CMC-Oat)).

### 2.2. Carboxylation of Cellulose

**Figure 1:**
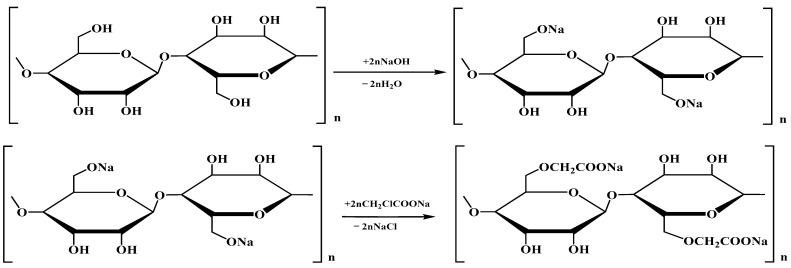
Reaction of cellulose carboxylation.

The carboxylation reaction was carried out in two stages (Figure 1). At the first stage, the reaction mass consisting of PC-Oat (5 g), isopropyl alcohol (50 mL) and NaOH (4.1 g) was activated by exposure to microwave radiation of different powers (210–350 W) for 30–120 s. Monochloroacetic acid (6.9 g) was added to the activated reaction mass and the process was continued under the same conditions. Then, the precipitate was separated in a Buechner funnel and washed with 70% aqueous ethanol solution. The obtained product was filtered in a vacuum filter, and then dried in air.

### 2.3. Preparation of Elastomer Composites

Preparation of the elastomer composites was carried out in two stages. At the first stage, ingredients were mixed by a laboratory two-roll mill without a vulcanizing agent and swelling filler (phr): BNKS-28 AMN (100.0), (JSC “Krasnoyarsk Synthetic Rubber Plant”, Krasnoyarsk, Russia); ZnO (5.0), (LLC “Empils-zinc”, Rostov-on-Don, Russia); stearic (2.0), (JSC “Nefis Cosmetics”, Kazan, Russia); 2-mercaptobenzothiazole (0.8), (JSC “Volzhsky Orgsintez”, Volgagrad, Russia); carbon black (45.0), (JSC “Sterlitamak Petrochemical Plant”, Sterlitamak, Russia).

The rubber compound was kept for 1 day at room temperature. In the second step, the rubber compound was mixed with water-swelling filler, vulcanizing agent (sulfur, CJSC “SERA”, Orenburg, Russia) and plasticizer (a number of samples) in the closed laboratory rubber mixer of a “Plasti-Corder^®^ Lab-Station” W50 E (Brabender, Duisburg, Germany). Oxal T-92 (LLC PKF “Khimavangard” Dzerzhinsk, Russia), which is a product of the additional processing of high-boiling by-products of dimethyldioxane production, was used as a plasticizer. The plasticizer was entered in the ratio of 30 parts per 100 parts of BNKS-28 AMN.

### 2.4. Measurements

#### 2.4.1. Fourier Transform Infrared Spectroscopy

Functional group analysis of the initial powdered lignocellulose (PC-Oat) and carboxylated powdered lignocellulose from oat straw (CMC-Oat) was carried out using the IR-Fourier spectrometer «Nicolet iS10» (Thermo Fisher Scientific, Waltham, MA, USA) [48]. The measurements were carried out in the range of 600 to 4000 cm^−1^, and the spectrum resolution was 2 cm^−1^.

#### 2.4.2. Thermogravimetric Analysis (TGA)

The thermal stability of lignocellulose (PC-Oat) and carboxylated lignocellulose (CMC-Oat) was evaluated using a STA 6000 thermogravimetric analyzer (PerkinElmer, Waltham, MA, USA) at a heating rate of 5 °C/min in the temperature range of 30–500 °C.

#### 2.4.3. Hildebrand–Scatchard Solubility Parameter Calculation

Solubility parameters δ were calculated using the Hildebrand–Scatchard [49] method using the equation:

$$\delta =\frac{\sum ∆E_{i}^{*}}{N_{A}\times \sum {∆V}_{i}},$$

where ∆E_i_* is the contribution of each atom and type of intermolecular interaction to the cohesive energy of the liquid, reduced by as many times as the van der Waals volume of the molecule is less than the molar volume; ∆*V_i_* is the van der Waals volume of repeating links of components; *N_A_* is the Avogadro constant, mol^−1^.

Thermodynamic compatibility is calculated using the formula [50]:

*β* = (*δ_rubber_* − *δ_component_*)^2^

#### 2.4.4. Scanning Electron Microscope (SEM)

The distribution of water-swelling fillers and the morphology of the rubber compounds were investigated on a scanning electron microscope (SEM) JSM-7800F (Jeol, Akishima, Japan) in the secondary electron mode at an accelerating voltage of 1.0–1.5 kV. The samples for the study (brittle chips) were obtained by the brittle fracture method at liquid nitrogen temperature.

#### 2.4.5. Determination of Curing Optimum, Vulcanization and Physical-Mechanical Tests

The rheometric characteristics of rubber compounds were determined on a rheometer «Monsanto 100 S» (Monsanto, St. Louis, MO, USA) at 160 °C. For determination of the tensile strength (TS), elongation at break was carried out using a tensile testing machine RMI-250 (POLIMERMASH GROUP, St. Petersburg, Russia), (sample stretching speed 500 mm/min). Tensile strength (*TS*) was determined using the formula:

$$TS=\frac{P_{0}}{{d\times m}_{0}},\;MPa$$

where *P_b_* is the force at break, H; *d* is the mean value of the sample thickness before the test, m; *b*_0_ is the sample width before the test, m.

#### 2.4.6. Determination of Viscosity by Mooney

A Mooney Viscozimeter-UGT7080S2 GOTECH (Taichung, Taiwan) determined the viscosity of the rubber at a temperature of 100 °C (material preheating period 1 min, rotor rotation duration 4 min).

#### 2.4.7. Determination of Sorption Properties

The degree of rubber swelling was determined by the change in the mass of the sample using the formula:

$$\alpha =\frac{m-m_{0}}{m_{0}}\times 100\%,$$

where *m* is the mass of the swollen sample, and *m*_0_ is the mass of the original sample.

The degree of rubber swelling was also determined by changing the volume of the sample according to the formula:

$$\alpha =\frac{V-V_{0}}{V_{0}}\times 100\%,$$

where *V* is the volume of the swollen sample, and *V*_0_ is the volume of the original sample. Model aqueous solutions of various mineralization (Table 1) and sodium chloride formation water were used for testing.

## 3. Results and Discussion

The selection of rubber fillers and plasticizers was based on rubber compatibility. It is most favorable if the enthalpy of mixing tends to zero, which is possible with the maximum proximity of the solubility parameters (*δ*) of the mixing components characterizing the compatibility parameter (*β*). The solubility parameters of BNKS-28 AMN, cellulose, Na-CMC and Oxal T-92 were calculated using the Hildebrand–Scatchard equation (Table 2). These components have been found to have thermodynamic compatibility (*β* < 0.5 MJ/m^3^). This indicates that a high degree of filling of the rubber matrix with these ingredients is possible.

**Figure 2:**
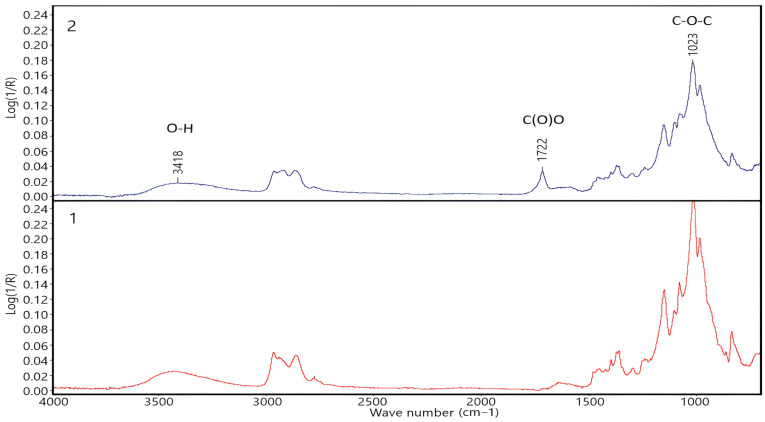
IR spectra (reflection spectra): 1—powdered lignocellulose from oat straw (PC-Oat), 2—carboxylated product of lignocellulose from oat straw (CMC-Oat).

The presence of >C=O groups in the product of carboxylation of lignocellulose from oat straw was recorded by Fourier transform infrared spectroscopy. The measurements were carried out in the range of 600 to 4000 cm^−1^. The IR spectrum of the carboxylated product of CMC-Oat (Figure 2, graph 2) showed a broad absorption band of valence vibrations of O–H bonds involved in the formation of inter- and intramolecular hydrogen bonds with a maximum at 3418 cm^−1^, a band of valence vibrations characteristic of the carboxyl group >C=O with a peak maximum at 1722 cm^−1^ and a band of νa vibrations of the simple ether bond (C–O–C) in the region of 1023 cm^−1^.

**Figure 3:**
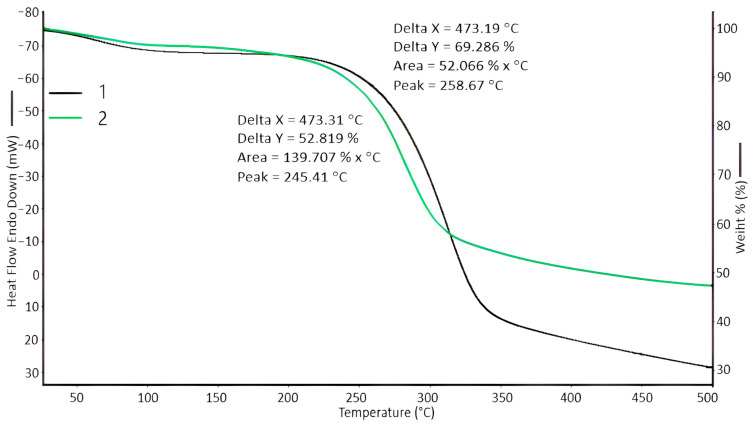
TGA curves: 1—powdered lignocellulose from oat straw (PC-Oat), 2—carboxylated product of lignocellulose from oat straw (CMC-Oat).

The thermal stability of the swelling fillers was evaluated by thermogravimetry (Figure 3). It was found that PC-Oat and the carboxylated product, CMC-Oat, when heated to 140 °C, lose 5.3% of their physically related water.

The mass loss of the sample PC-Oat starts at 259 °C and ends at 380 °C. During intensive decay, about 55% of the mass of the initial sample is lost. Then, the interval from 380 to 500 °C exhibits a slow mass reduction; in this interval, about 10% of the mass of the sample is lost, and the carbonized residue at 500 °C is 30%. The CMC-Oat, unlike the PC-Oat, loses 35% of the mass in a rapid stage in the range of 260–360 °C. Then, in the interval 360–500 °C, it exhibits a slow mass reduction; in this interval, about 10% of the mass of the sample is lost, and the carbonized residue at 500 °C is 47%.

**Figure 4:**
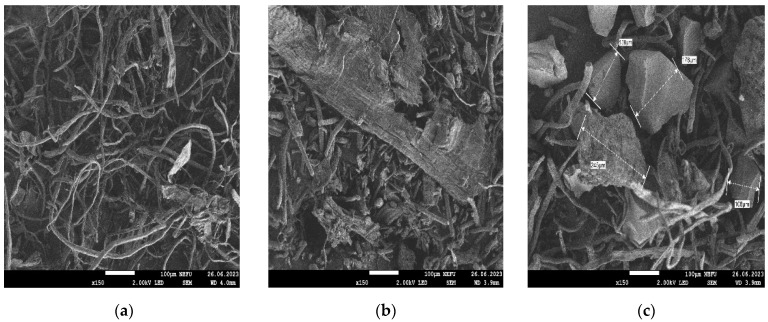
Microphotographs of samples of swelling fillers: (**a**) PC-Oat, (**b**) CMC-Oat, (**c**) Na-CMC.

Figure 4a shows an image of the original lignocellulose (PC-Oat), which was obtained by alkaline hydrolysis. This sample has a fibrous structure, where the fiber width is about 20/50 mm and the length is 0.5/1.5 mm. Figure 4b shows a photograph of partially carboxylated lignocellulose (CMC-Oat) obtained from PC-Oat. The sample also has a fibrous structure, but the fiber length is reduced. Figure 4c shows a photograph of a commercial sample of the sodium salt of carboxymethylcellulose (Na-CMC). The sample with the fibers has large inclusions, the size of which ranges from 130 to 250 mm.

The swelling filler was introduced in the ratio of BRC/SF = 1:1 by weight (Table 3). The plasticizer was used in a number of samples. The composition of the swelling filler varied during the study.

The analysis of the vulcanization rheograms of rubber compounds presented in Table 4 led to the following conclusions. Replacement of Na-CMC with PC-Oat resulted in a significant increase in the minimum torque (M_min_) characterizing the viscosity of rubber compounds. This could be due to stronger hydrogen bonds formed due to OH groups of PC-Oat compared to hydrogen bonds formed due to carboxyl groups of Na-CMC. This statement was confirmed by the fact that after partial carboxylation of PC-Oat, the M_min_ decreased to the level of the control sample without swelling filler. This indicates that by varying the degree of carboxylation, it is possible to control the viscosity characteristics of rubber compounds including swelling filler from PC-Oat and its carboxylated product. Maximum torque (M_max_) was also most significantly increased for the sample containing the PC-Oat filler. Maximum torque enhancement is not desirable as it may have a negative effect on the processing of the rubber compound. In practice, plasticizers are used to reduce M_max_. The plasticizer molecules penetrate between the polymer chains, reducing intermolecular forces while increasing macromolecular mobility. Reducing the viscosity of rubber compounds using plasticizers can have several positive effects. First, reducing viscosity facilitates the distribution of ingredients in the compound. This is particularly important because the uniform distribution of ingredients affects the quality and properties of the final product. Secondly, plasticizers increase the plasticity of the rubber compound, making it easier to process. When selecting a plasticizer for use in rubber compounds, its properties should be taken into account. In this work, the polar product Oxal T-92 was used as a plasticizer because it is compatible with polar rubbers, the group to which BNKS-28 AMN belongs, and also with polar swellable filler (Table 4). Based on the analysis of the rheometric vulcanization curves, the optimal time for vulcanization of the rubber compounds at 160 °C was 20 min.

The data from the Mooney viscosity measurements of the rubber compounds agree with the data of torque determination (Table 5). According to the literature data, as the Mooney viscosity increases, its plasticity decreases, and uniform distribution of ingredients becomes more difficult [51]. When the plasticizer was applied, the viscosity of Mooney was reduced by 10–15%.

**Figure 5:**
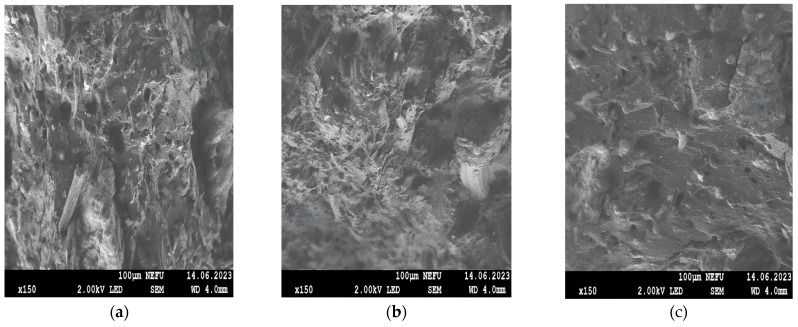
Microphotographs of the samples: (**a**) PC-Oat, (**b**) CMC-Oat, (**c**) Na-CMC. Microphotographs of plasticized rubber samples filled with (Respectively).

The morphology of chips from the rubber sample surfaces was investigated by SEM. When using PC-Oat (Figure 5a), uneven distribution of the filler, the formation of heterogeneous morphology and the presence of micropores are observed. When PC-Oat is carboxylated (Figure 5b), the phase boundary between the CMC-Oat and rubber particles is blurred, which is probably due to the mutual diffusion of particles when smaller filler particles are melted. The micropores are preserved. The rubber sample containing Na-CMC (Figure 5c) is characterized by a structure with a large number of pores in which the Na-CMC filler is distributed randomly. The size of Na-CMC phases in the volume varies widely. The sample shows weak interphase interaction between the swelling filler and the binder, which probably leads to a decrease in the strength of the vulcanized rubber (Table 5) and an increase in its swelling ability.

The introduction of Na-CMC led to a sharp reduction in the tensile strength of the rubber (*TS*) compared to the control sample without swelling fillers (Table 6), which was previously recorded in a number of studies [32]. Apparently, Na-CMC changes the structure of the cross-linked rubber network, which leads to disruption of the internal interactions and weak adhesion in the material. This may cause mechanical instability and weak bonding between polymer chains, which negatively affects the tensile strength of the rubber. The use of carboxylated CMC-Oat increased the *TS* by 14%, whereas when Na-CMC was replaced by PC-Oat, the *TS* doubled. When plasticizer T-92 was introduced into the rubber composition, the elongation at break (ε) increased from 10% to 60% in comparison with samples without the plasticizer.

**Figure 6:**
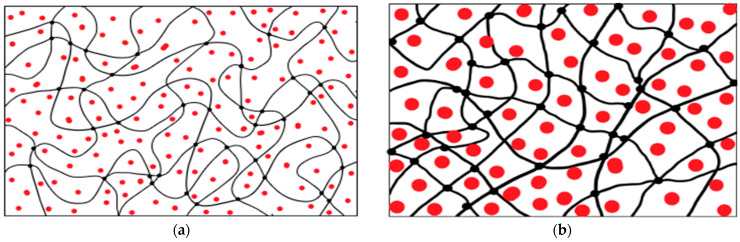
Visualization of the swelling process of samples: (**a**)—before swelling, (**b**)—during swelling.

To characterize the degree of swelling of cellulose material, swelling when changing the mass and when changing the volume are determined. Rubber swelling tests were carried out in model aqueous sodium/calcium/chloride solutions with different degrees of mineralization (Figure 6).

There are various theories explaining the swelling of cellulose and carboxylated cellulose, but all of them do not fully reflect this complex phenomenon [52,53]. Therefore, the mechanism of swelling cannot be considered conclusively clarified.

According to the hydration theory, the cause of the strong swelling of cellulose in solutions of electrolytes, including alkalis, is the selective adsorption by cellulose of one of the ions of the electrolyte, which carries an aqueous envelope with it. The cellulose surface, attracting hydrated ions to itself, is enriched with water.

In terms of this theory, the different degrees of swelling of cellulose in aqueous solutions are due to the different degrees of hydration of their ions. The swelling of cellulose is more intense when the cation hydration degree and the size of the anion are increased. According to the degree of hydration, the ions are arranged in the following series:

Li^+^ > Na^+^ > K^+^ > Rb^+^ > Cs^+^ > Zn^2+^ > Mg^2+^ > Ca^2+^ > Ba^2+^ > I^−^ > Br^−^ > Cl^−^ > F^−^~SO_4_^2−^.

Based on the fact that Na-CMC already has Na^+^ cations attracting the solvate envelope, the degree of swelling of the rubber with Na-CMC is expectedly higher than that of the rubber with CMC-Oat. In concentrated solutions, the degree of swelling of cellulose decreases, which is explained by a decrease in the degree of hydration of cations.

**Figure 7:**
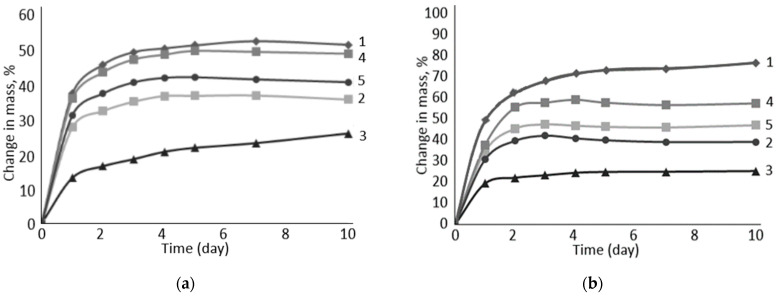
Influence of water-swelling filler composition on the degree of rubber swelling: 1—Na-CMC, 2—Na-CMC + PC-Oat, 3—PC-Oat, 4—Na-CMC + CMC-Oat, 5—CMC-Oat. Medium: (**a**)—I, (**b**)—II.

The experiment showed that vulcanized rubber filled with the carboxylated form of cellulose is most sensitive to the degree of salinity of the aqueous solution (Figure 7). In low mineralization (II) in the model sodium/calcium/chloride solution, the degree of swelling of such rubber is higher than in the high-mineralization solution (I). The decrease is about 10%. At the same time, there is a general trend of decrease in the degree of swelling during the transition from Na-CMC to PC-Oat. This suggests that carboxyl groups, which are stronger electrolytes than alcohol groups, play the most important role in the rubber swelling process.

**Figure 8:**
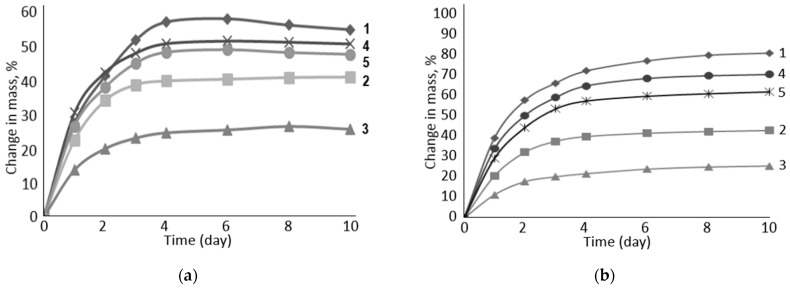
Influence of water-swelling filler composition on the degree of rubber swelling: 1—Na-CMC, 2—Na-CMC + PC-Oat, 3—PC-Oat, 4—Na-CMC + CMC-Oat, 5—CMC-Oat. Medium: (**a**)—III, (**b**)—IV.

The data shown in Figure 8 indicate that the rubber used in sodium chloride formation water exhibits a similar swelling degree dependence as described earlier in the model system in Figure 7.

**Figure 9:**
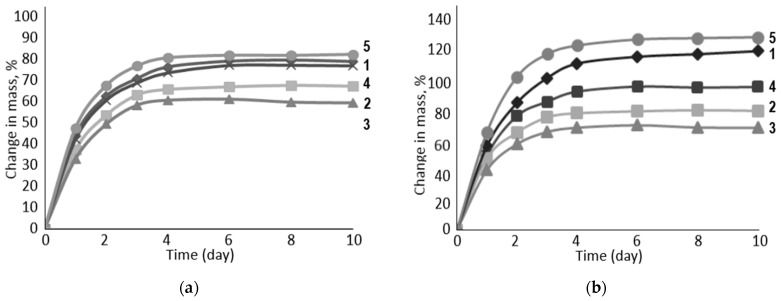
Influence of water-swelling filler composition on the degree of rubber swelling: 1—Na-CMC, 2—Na-CMC + PC-Oat, 3—PC-Oat, 4—Na-CMC + CMC-Oat, 5—CMC-Oat. Medium: (**a**)—V, (**b**)—VI.

Based on the fact that in the model system (Figure 9), which uses NaOH aqueous solution, the rubber samples including PC-Oat had a fairly high degree of swelling, the composition of the electrolyte anion is expected to have a significant impact on the degree of swelling.

**Figure 10:**
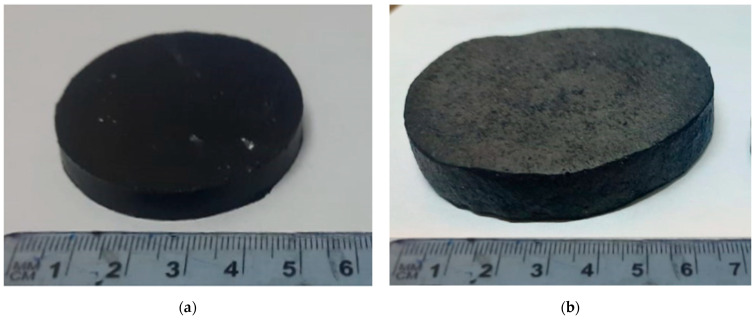
Photographs of a disk-shaped rubber sample: (**a**) before swelling, (**b**) swollen in 5% NaOH solution.

The samples for measuring the volume of rubbers during swelling in aqueous media were disks with a diameter of 50 mm and a height of 6 mm (Figure 10a). Figure 10b shows the same sample filled with CMC-Oat after swelling in an alkaline medium. The sample swelled uniformly in three directions.

Based on the data presented in Table 7, it can be seen that when rubber swells, the increase in volume exceeds the increase in mass by 10–12%. In particular, in model sodium chloride solutions, the samples filled with CMC-Oat are characterized by a degree of swelling, measured by a change in mass of 40% and 45%, while the change in volume is 45% and 51%, respectively. In alkaline solutions, these values were 82% and 121% (mass change) and 91% and 134% (volume change), respectively.

## 4. Conclusions

Carboxylation of powdered lignocellulose from oat straw was carried out. Microwave radiation was used to activate the carboxylation process. The carboxylated product has been tested as a swelling filler in the composition of a highly filled rubber with a sulfur vulcanization system based on nitrile butadiene rubber grade BNKS-28 AMN. Sodium carboxymethylcellulose is used as a comparison filler. It is shown that the carboxylation of powdered lignocellulose from oat straw makes it possible to increase the swelling degree of rubber in aqueous solutions of various mineralization in comparison with noncarboxylated lignocellulose. A fairly high degree of rubber swelling and an increase in the strength characteristics of rubber were noted when using mixed swelling fillers of sodium carboxymethylcellulose with partially carboxylated lignocellulose from oat straw.

## Figures and Tables

**Table 1:** Composition of aqueous solutions of various mineralizations for testing the adsorption capacity of rubbers.

	Name of Aqueous Solution	Ion Content, g/L					Density, kg/m^3^
		Na^+^	Ca^2+^	Mg^2+^	Cl^−^	Total	
I	Model sodium chloride solution	59	11	-	110	180	1100
II	Model sodium chloride solution	26	12	-	62	100	1060
III	Formation water (sodium chloride)	70	11	3	139	223	1157
IV	Formation water (sodium chloride)	34	5	2	59	100	1070
V	NaOH 10%	57.5	-	-	-	57.5	1108
VI	NaOH 5%	28.8	-	-	-	28.8	1054

**Table 2:** Solubility and compatibility parameters of the used rubber compound components.

Component	Cohesive Energy	Van der Waals Volume	Solubility Parameter	Compatibility of the Components with BNKS-28 AMN
	Δ*Ε_i_*, J/mol	*N_A_*Ʃ*V_i_* × 10^6^, m^3^/mol	*δ*, (MJ/m^3^)^1/2^	*β*, MJ/m^3^
BNKS-28 AMN	12,123	61.4	18.0	-
Cellulose	16,543	85.3	17.6	0.16
Na-CMC	21,639	117.4	17.8	0.04
Oxal T-92	20,404	96.1	18.7	0.49

**Table 3:** Compositions of swelling rubber.

Sample No	Quantity, Mass Fraction, %			
	BRC	Na-CMC	PC-Oat	CMC-Oat
Without plasticizer				
1	100	-	-	-
2	50	50	-	-
3	50	25	25	-
4	50	-	50	-
5	50	25	-	25
6	50	-	-	50
Plasticizer T-92 (30 phr)				
7	100	-	-	-
8	50	50	-	-
9	50	25	25	-
10	50	-	50	-
11	50	25	-	25
12	50	-	-	50

**Table 4:** Parameters of rheometric vulcanization curves of rubber compounds («Monsanto 100S», ratio BRC/SF = 1:1 by mass).

Composition of Rubber Compounds (Ratio, Mass Fraction, %)		t_s_, min	M_min_, dN·m	M_max_, dN·m	t_90_, min
Without plasticizer					
1	Control sample (BRC)	1.2	24	54	19.8
2	BRC/Na-CMC (50: 50)	1.5	26	60	18.8
3	BRC/PC-Oat (50:50)	1.7	35	67	17.6
4	BRC/CMC-Oat (50:50)	1.8	24	53	17.4
5	BRC/Na-CMC/PC-Oat (50:25:25)	1.6	28	62	18.1
6	BRC/Na-CMC/CMC-Oat (50:25:25)	1.6	26	56	18.2
Plasticizer T-92 (30 phr)					
7	Control sample (BRC)	1.8	18	42	18.3
8	BRC/Na-CMC (50:50)	2.1	17	40	18.6
9	BRC/PC-Oat (50:50)	2.5	20	49	17.3
10	BRC/CMC-Oat (50:50)	2.4	14	36	17.6
11	BRC/Na-CMC/PC-Oat (50:25:25)	2.3	18	44	17.8
12	BRC/Na-CMC/CMC-Oat (50:25:25)	2.2	15	37	18.8

**Table 5:** Analysis of the results of Mooney viscosity.

Indicator	Swelling Filler					
	–(Control without SF)	Na-CMC	PC-Oat	CMC-Oat	Na-CMC + PC-Oat	Na-CMC + CMC-Oat
	1	2	3	4	5	6
Mooney Viscosity ML(1 + 4) 100 °C	48.7	74.6	180.6	62.2	104.9	75.3
Plasticizer T-92 (30 phr)						
	7	8	9	10	11	12
Mooney Viscosity ML(1 + 4) 100 °C	43.5	64.3	160.1	55.4	96.3	67.8

**Table 6:** Physical and mechanical properties of rubbers.

Properties	Swelling Filler					
	–(Control without SF)	Na-CMC	PC-Oat	CMC-Oat	Na-CMC + PC-Oat	Na-CMC + CMC-Oat
Without plasticizer						
	1	2	3	4	5	6
*TS*, MPa	13.8	3.6	8.6	4.1	5.3	4.6
ε, %	400	310	30	320	70	280
Plasticizer T-92						
	7	8	9	10	11	12
*TS*, MPa	14.8	3.4	7.2	3.9	4.8	4.3
ε, %	480	340	50	350	90	320

**Table 7:** Volume change data in aqueous media.

N	Swelling Filler	Aqueous Media (Table 1)					
		I	II	III	IV	V	VI
		Volume Change in the Samples, %					
1	Na-CMC	59	94	61	86	84	125
2	Na-CMC + PC-Oat	39	45	43	47	73	88
3	PC-Oat	26	28	24	29	64	77
4	Na-CMC + CMC-Oat	54	63	57	76	87	107
5	CMC-Oat	45	51	52	66	91	134

## Data Availability

Data are contained within the article.
